# Supplementary material for: Megafaunal extinctions, not climate change, may explain Holocene genetic diversity declines in Numenius shorebirds
Source: eLife. 2023 Aug 7;12:e85422. doi: 10.7554/eLife.85422 (PMC10406428; doi:10.7554/eLife.85422)
Supplement: Supplementary file 2. — Legend: Evolutionary distinctness, phylogenetic diversity, and evolutionarily distinct and globally endangered (EDGE) scores of Numenius species. [file elife-85422-supp2.docx]

Evolutionary distinctness, phylogenetic diversity and evolutionarily distinct and globally endangered (EDGE) scores of Numenius species, as calculated from MCMCTree branch lengths and IUCN status (Jetz et al., 2014). IUCN (2020) status abbreviations: CR – Critically Endangered; EN – Endangered; NT – Near Threatened; LC – Least Concern. Asterisks (*) given for putatively extinct species.

| **Clade** | **Species** | **IUCN Status** | **Evolutionary distinctness in million years (MY)** | **Phylogenetic diversity (my)** | | **EDGE score** |
| --- | --- | --- | --- | --- | --- | --- |
| whimbrels | *N. tahitiensis* | NT | 3.18 | 12.23 | 25.10 | 2.12 |
|  | *N. phaeopus* | LC | 2.59 |  |  | 1.28 |
|  | *N. hudsonicus* | LC | 2.59 |  |  | 1.28 |
|  | *N. minutus* | LC | 3.87 |  |  | 1.58 |
| curlews | *N. tenuirostris* | CR* | 2.31 | 12.87 |  | 3.97 |
|  | *N. arquata* | NT | 2.31 |  |  | 1.89 |
|  | *N. madagascariensis* | EN | 2.43 |  |  | 3.31 |
|  | *N. americanus* | LC | 2.91 |  |  | 1.36 |
|  | *N. borealis* | CR* | 2.91 |  |  | 4.14 |

# **References**

IUCN. (2020). The IUCN Red List of Threatened Species. Version 2020-3. Retrieved from https://www.iucnredlist.org

Jetz, W., Thomas, G. H., Joy, J. B., Redding, D. W., Hartmann, K., & Mooers, A. O. (2014). Global distribution and conservation of evolutionary distinctness in birds. *Current Biology*, *24*(9), 919–930. https://doi.org/10.1016/j.cub.2014.03.011
