# Supplementary material for: Megafaunal extinctions, not climate change, may explain Holocene genetic diversity declines in Numenius shorebirds
Source: eLife. 2023 Aug 7;12:e85422. doi: 10.7554/eLife.85422 (PMC10406428; doi:10.7554/eLife.85422)
Supplement: Supplementary file 3. — Legend: Details of occurrence points, parameters, and results of ecological niche modeling using Maxent. [file elife-85422-supp3.docx]

Section A Details of occurrence points in breeding areas used for input into Maxent for four Numenius species, including sample size, month (x denotes the month for which records are included), year and sources of records.

| **Species** | **Number of samples** | **Month** | | | | **Year** | **Source** |
| --- | --- | --- | --- | --- | --- | --- | --- |
|  |  | **Apr** | **May** | **Jun** | **Jul** |  |  |
| *N. phaeopus* | 158 |  | x | x | x | All years | eBird, 2021; GBIF.org, 2022a; Lappo, Tomkovich, & Syroeckovskiy, 2012 |
| *N. hudsonicus* | 1713 |  | x | x | x | All years | eBird, 2021; GBIF.org, 2022b, 2022c, 2022d |
| *N. americanus* | 644 | x | x | x |  | 1960 – 1990 | eBird, 2021; GBIF.org, 2022e |
| *N. arquata* | 997 | x | x | x | x | All years | eBird, 2021; GBIF.org, 2022f |

Section B. Summary of the parameters and results of the best ecological niche model identified for each of the four Numenius target species.

| **Species** | **Feature class(es)** | **Regularisation multiplier** | **Mean validation area under the receiver operating curve (auc.val.avg)** | **Mean validation continuous Boyce index (cbi.val.avg)** | **Mean minimum training presence omission rate (or.mtp.avg)** | **Maximum test sensitivity plus specificity Cloglog threshold** |
| --- | --- | --- | --- | --- | --- | --- |
| *N. phaeopus* | LQHP | 4 | 0.831 | 0.836 | 0.006 | 0.570 |
| *N. hudsonicus* | LQH | 2 | 0.829 | 0.691 | 0.044 | 0.408 |
| *N. americanus* | LQ | 4 | 0.881 | 0.733 | 0.002 | 0.459 |
| *N. arquata* | L | 0.5 | 0.827 | 0.757 | 0.002 | 0.477 |

# **References**

eBird. (2021). eBird Basic Dataset. Version: EBD_relNov-2021. Ithaca, New York: Cornell Lab of Ornithology.

GBIF.org. (2022a). GBIF Occurrence Download. Retrieved May 19, 2022, from https://doi.org/10.15468/dl.xuxpvq

GBIF.org. (2022b). GBIF Occurrence Download. Retrieved April 27, 2022, from https://doi.org/10.15468/dl.sybhg8

GBIF.org. (2022c). GBIF Occurrence Download. Retrieved April 27, 2022, from https://doi.org/10.15468/dl.e8jkmw

GBIF.org. (2022d). GBIF Occurrence Download. Retrieved April 27, 2022, from https://doi.org/10.15468/dl.s8g354

GBIF.org. (2022e). GBIF Occurrence Download. Retrieved April 27, 2022, from https://doi.org/10.15468/dl.c8xvcq

GBIF.org. (2022f). GBIF Occurrence Download. Retrieved May 11, 2022, from https://doi.org/10.15468/dl.7fs5mn

Gill, F., Donsker, D., & Rasmussen, P. (Eds.). (2021). *IOC World Bird List (v11.1)*. https://doi.org/10.14344/IOC.ML.11.1.

Lappo, E. G., Tomkovich, P. S., & Syroeckovskiy, E. E. (2012). *Atlas of breeding waders in the Russian Arctic*. Moscow: Institute of Geography, Russian Academy of Sciences.
